# Emotional Regulation and Risk of Eating Disorders in Adolescent Athletes

**DOI:** 10.3390/ejihpe15090188

**Published:** 2025-09-18

**Authors:** Silvia P. Espinoza-Barrón, Abril Cantú-Berrueto, María Á. Castejón, Rosendo Berengüí

**Affiliations:** 1 Faculty of Sports Organization, Autonomous University of Nuevo León, San Nicolás de los Garza 66455, Mexico; silvia.espinozaba@uanl.edu.mx (S.P.E.-B.); abril.cantubrr@uanl.edu.mx (A.C.-B.); 2 Faculty of Education, Universidad Católica de Murcia (UCAM), 30107 Murcia, Spain; macastejon@ucam.edu

**Keywords:** emotional regulation, eating disorders, youth sports, risk factors, psychometric assessment

## Abstract

Eating Disorders (EDs) are more prevalent among athletes due to performance pressure and body ideals. Emotional regulation is a key factor in ED. This study aimed to (1) examine the reliability and structural validity of the Emotion Regulation Questionnaire for Children and Adolescents (ERQ-CA) in adolescent Mexican athletes, and (2) analyze associations between emotional regulation strategies (expressive suppression and cognitive reappraisal) and ED risk factors (drive for thinness, body dissatisfaction, and bulimia). An instrumental, cross-sectional design was employed with 295 Mexican athletes (*M*age = 16.85, *SD* = 3.27). The ERQ-CA demonstrated good psychometric properties, with acceptable reliability (*ω* > 0.70) and excellent fit for the two-factor model (CFI = 0.995, RMSEA = 0.018). Emotional suppression was positively associated with all ED risk indicators, whereas cognitive reappraisal was negatively associated. These findings highlight that individual differences in emotion regulation are linked to ED risk in adolescent athletes. Monitoring expressive suppression and promoting cognitive reappraisal may serve as supportive strategies for coaches, parents, and mental health professionals, enhancing emotional flexibility and potentially reducing ED risk.

## 1. Introduction

Eating disorders (EDs) are a group of diseases that cause distorted eating behavior and extreme concern about self-image and body weight, leading to harmful weight control behaviors such as vigorous physical activity, food restriction, fasting, and abuse of laxatives and diuretics (Garner & Garfinkel, 1979; Gouveia et al., 2010). This ultimately leads to a significant deterioration in the individual’s physical, psychological, and social health (Attia & Walsh, 2022; Berengüí et al., 2016).

According to the Diagnostic and Statistical Manual of Mental Disorders (DSM-5) of the American Psychiatric Association (2013), EDs include anorexia nervosa, bulimia nervosa, binge eating disorder, pica, rumination, and restricted eating disorder, avoidant/restrictive food intake disorder, and eating disorder specified and unspecified.

Several studies have identified a higher prevalence of ED in athletes compared to the general population. This risk is particularly high in aesthetic disciplines (such as gymnastics, figure skating, and dance), followed by weight-class sports (such as wrestling or combat sports), gym sports (such as fitness or bodybuilding), and finally endurance sports (such as track and field, cycling, and swimming) (Aleksić et al., 2020; Berengüí et al., 2024; Daley et al., 2023; Purcell et al., 2019).

Some of the factors that contribute to this problem are social pressure from coaches and teammates, body weight requirements for competition, perfectionism, athletic identity, gender, competitive level, coaching leadership styles, and age (Hernández-Mulero & Berengüí, 2016; Teixidor Batlle et al., 2019).

These risks are accentuated during adolescence, due to the important biological, social and psychological changes that occur during this stage (Crone, 2016). It is a period of special significance, as adolescents receive multiple influences that affect personality development and self-concept, increasing mental health vulnerability (Parise et al., 2019; Pérez-Sánchez et al., 2024; Slobodskaya, 2021). This susceptibility is related to factors such as brain immaturity, hormonal changes and exposure to contexts that hinder decision-making and behavior regulation. The situation is exacerbated in adolescents who simultaneously face the demands of competitive sport and are exposed to potentially stressful contexts. This makes them more prone to problems such as behavioral disorders, ED and emotional disorders (World Health Organization, 2025; Pan American Health Organisation, 2025; UNICEF, 2020)

ED can go unnoticed in athletes for several reasons. These include inattention to signs and symptoms, maintaining a normal weight with very low fat, excessive exercise as compensatory behavior, and failure to recognize physiological indicators such as amenorrhea, decreased performance, or nutritional deficiencies (Daley et al., 2023). On the other hand, ED has been linked to difficulties in emotional regulation processes. It is estimated that between 40% and 75% of people with eating disorders face significant challenges in this area, such as difficulties in managing emotions, distorted perceptions of behavior, and the use of maladaptive strategies (Gross & Jazaieri, 2014; Malagoli et al., 2021; Meule et al., 2021).

Emotional regulation is defined as a complex process that involves a person’s ability to manage and influence their own emotions, both when they arise and in the way they are experienced and expressed (Gross, 1998; Thompson, 1994; Vargas Gutiérrez & Muñoz-Martínez, 2013). According to Gross’s (1998) model of the emotional regulation process, people use two types of strategies for emotional management. These can be focused on antecedents (before the emotion is fully activated) or on the response (when the emotion has already been triggered).

Among the most studied strategies are Cognitive Reappraisal (CR) and Expressive Suppression (ES). CR is a cognitive strategy focused on the antecedent, which involves reinterpreting a situation to alter its emotional impact. For example, instead of thinking ‘this is terrible,’ one might think ‘this is a challenge I can overcome.’ ES, on the other hand, is a response-focused strategy that involves hiding, inhibiting, or reducing emotional expressive behavior. For example, an athlete who suppresses their anger may show an expressionless face, even though they are feeling very angry (Cutuli, 2014; Pastor et al., 2019).

The use of each of these strategies has been associated with different consequences. While CR has been associated with adaptive and well-being effects, ES has been linked to greater negative effects and a variety of mental disorders, including depression, anxiety and ED (Freire et al., 2020; Gross & Jazaieri, 2014; Mikulic, 2021).

Recent systematic reviews and empirical studies consistently highlight the crucial role of emotional regulation in both athletic performance and mental health. Evidence shows that an athlete’s ability to monitor and manage their emotions adaptively through strategies like CR is linked to enhanced concentration, resilience, and better sport-specific performance in individual and team contexts. In contrast, maladaptive strategies such as ES are associated with increased psychological distress, reduced well-being, and poorer performance outcomes (Bird et al., 2021; Boas Junior et al., 2025; Martínez-Líbano et al., 2025).

In competitive environments, emotional regulation interacts with environmental, social, and psychological factors, influencing physiological responses (e.g., heart rate and cortisol levels) and cognitive states (Boas Junior et al., 2025). Furthermore, difficulties in this process have been directly linked to higher symptoms of depression and anxiety among competitive athletes. Preliminary evidence suggests that interventions targeting emotional regulation can reduce emotional dysregulation and improve performance satisfaction (Tamminen et al., 2025).

Similar patterns have emerged in newer sports, such as esports, where players report performance-impairing emotional experiences and a lack of effective coping mechanisms (Beres et al., 2023). In sum, these results highlight the importance of emotion regulation in performance environments where the risk of EDs is higher.

The high prevalence of difficulties in emotional regulation in ED highlights the need to deepen our understanding of this factor in their development and maintenance, to identify risk and protective factors. This need becomes even more relevant in the sports field, where emotional demands, performance pressure, and body ideals can intensify the vulnerability of athletes (Berengüí et al., 2024; Da Costa Dutra et al., 2023).

In this regard, it is essential to have valid and reliable instruments that allow for the evaluation of emotional regulation strategies in this specific population. The Emotion Regulation Questionnaire for Children and Adolescents (ERQ-CA) is one of the most used tools for this purpose, allowing for the assessment of these strategies in individuals aged 9 and older. Some studies have also used the ERQ-CA in athletic populations (Kim & Tamminen, 2023; Ros-Morente et al., 2022). Recent validations in contexts such as the US, Europe, and Asia have demonstrated a stable two-factor structure of cognitive reappraisal and expressive suppression, adequate internal consistency, test–retest reliability, and measurement invariance across gender, age, and cultural groups (Aune et al., 2025; Chen et al., 2023; Gong et al., 2022). These findings support the ERQ-CA’s cross-cultural applicability, yet no research has been identified that addresses its psychometric validation in sports contexts in Latin America.

### Aims and Hypotheses

Considering the close relationship between emotion regulation, athletic performance, and ED risk, alongside the current lack of validated instruments for athletic populations in Latin America, the present study aims to examine the reliability and structural validity of the ERQ-CA in young Mexican athletes, as well as to analyze the relationship between emotional regulation strategies (ES and CR) and risk factors associated with ED (drive for thinness, body dissatisfaction, and bulimia) in this population.

Based on previous research and theory, we propose the following hypotheses:

**H1:**  *The ERQ-CA will exhibit the expected two-factor structure corresponding to Cognitive Reappraisal and Expressive Suppression, with acceptable model fit indices defined a priori (χ^2^/df < 5; CFI/NNFI ≥ 0.90; RMSEA ≤ 0.08).*

**H2:**  *The factorial structure of the ERQ-CA will be invariant across sex and across stages of adolescence.*

**H3:**  *CR will be negatively associated with ED risk factors, whereas ES will be positively associated with these risk factors.*

Accordingly, hypotheses H1 and H2 represent the primary outcomes of the study, whereas H3 is considered secondary.

## 2. Materials and Methods

An instrumental investigation was conducted to analyze the psychometric properties of the instrument. A non-experimental, cross-sectional, comparative design was also used to examine the characteristics of the population based on the variables studied.

### 2.1. Participants and Procedures

The sample comprised 295 Mexican athletes (M = 129; F = 166) aged between 10 and 25 years (M = 16.91; SD = 3.21). Of these, 70.1% participated in team sports (cheerleading, American football and basketball) and 29.9% in individual sports (dance, gymnastics and figure skating). In terms of sports experience, 50.5% had more than 7 years, 10.4% had between 4 and 6 years, 16.8% had between 1 and 3 years, and 22.2% had less than 1 year. Most athletes had regional-level competition experience.

Participants were recruited via private and public sports clubs and schools. Invitations were shared directly with coaches and parents between March 2023 and November 2024. No reimbursement or incentives were offered for participation. The following inclusion and exclusion criteria were applied to determine eligibility for participation:

Inclusion criteria:

- Active membership in a sports team
- Training at least three times per week
- Age between 10 and 25 years, encompassing early, middle, and late adolescence.

Exclusion criteria:

- Recreational practice only
- Inconsistent training attendance
- Lack of parental or guardian consent for minors

To ensure that the sample could include participants from early adolescence to emerging adulthood, this age span was chosen conceptually following the developmental stages proposed by the American Academy of Pediatrics (American Academy of Pediatrics et al., 2019) and Sawyer et al. (2018), as athletes often remain under structured training and supervision beyond strict adolescence. To support this decision statistically, measurement invariance of the ERQ-CA across adolescence stages (early, middle, late) was examined. Results supporting invariance are presented in the results section.

A post-hoc sensitivity analysis indicated that, with N = 295, α = 0.05, and power = 0.80, the study was able to detect correlations of r ≥ 0.19 in bivariate analyses, or regression effects explaining f^2^ ≥ 0.03 (≈3% of the variance) in multiple regression models.

The study was conducted in accordance with the Declaration of Helsinki (World Medical Association, 2017). The study was submitted for prior evaluation and obtained approval from the CEIFOD Ethics Committee with registration number CONBIOETI-CA-19-CEI-002-20220418. Participation was voluntary and formalized by signing an informed consent form, either by the athletes themselves if they were over 18 years of age, or by their parents or guardians if they were minors. Prior to data collection, an informational meeting was held with parents or guardians to explain the study and obtain their consent. Subsequently, athletes whose parents or guardians had given consent were invited to receive study information and provide their own assent before participating. If either the parent/guardian or the athlete did not agree, the questionnaire was not administered. The questionnaires were administered in person using digital media via Google Forms, with a sports psychologist present to answer any questions.

### 2.2. Measures

To assess emotional regulation, the Emotion Regulation Questionnaire for Children and Adolescents (ERQ-CA; Gullone & Taffe, 2012) was used. The scale consists of 10 items belonging to two factors: Cognitive Reappraisal and Expressive Suppression. The items are evaluated on a Likert-type response scale ranging from (1) Strongly Disagree to (5) Strongly Agree. Representative examples of the items are “When I want to feel less intensely a negative emotion (such as sadness or anger), I change what I’m thinking” (CR) and “I keep my emotions to myself” (ES).

In addition, the Eating Disorder Inventory-3 Referral Form (EDI-3 RF; Garner, 2004) was used. The version adapted by Elosua et al. (2010), with norms for population in Spain and Mexico, was employed. This instrument consists of 25 items that allow for screening for ED risk. It uses a Likert-type response scale ranging from (1) Never to (6) Always. Representative examples of the items include: “I eat or drink in secret” for the Bulimia factor, “I think my stomach is too big” for Body Dissatisfaction, and “If I gain a kilogram, I worry about continuing to gain weight” for Drive for Thin-ness. It is important to note that the EDI-3 RF is a standardized screening tool designed to identify individuals who may benefit from referral to specialized services; it does not provide a clinical diagnosis of an eating disorder.

### 2.3. Data Analysis

IBM SPSS 29, JASP 0.95 and G*Power 3.1.9.7 statistical software were used to analyze and process the results. Descriptive and normality statistics (mean, standard deviation, skewness and kurtosis) were calculated for the study variables to examine their distribution. To assess the internal consistency of the ERQ-CA, McDonald’s omega (*ω*) was calculated for both the total scale and its subscales (reappraisal and emotional suppression). Values between 0.60 and 0.80 were considered acceptable reliability and values greater than 0.80 were considered very good reliability (Dugard et al., 2010; Field, 2009; Streiner et al., 2015).

To examine the factorial validity of the instrument in the sports context, a Confirmatory Factor Analysis (CFA) was performed. The goodness of fit of the models took into account the following indices: chi-squared over degrees of freedom (*χ*^2^/*df*; Wheaton et al., 1977), considering values less than 5 as a good fit; the Non-Normed Fit Index (NNFI) and the Comparative Fit Index (CFI), where values greater than 0.90 were considered adequate; and the Root Mean Square Error of Approximation (RMSEA), where values below 0.08 were considered an acceptable fit (Hu & Bentler, 1995). Non-normal subscales (e.g., Bulimia) were analyzed using robust standard errors and supplemented with non-parametric checks to ensure convergent results.

Additionally, to assess measurement invariance, multigroup CFAs were conducted separately for sex (male and female) and age groups (early adolescence: 10–13 years; middle adolescence: 14–17 years; late adolescence: 18–25 years). A hierarchical sequence of nested models was tested, including configural, metric, scalar, and strict invariance. Model fit was evaluated using the same indices, and changes in CFI (ΔCFI ≤ 0.01) and RMSEA were used as criteria to determine invariance (Cheung & Rensvold, 2002).

To explore the relationship between emotional regulation and indicators of risk for ED, Pearson correlations were calculated, given the nature of the data. Student T tests were also applied to analyze differences in emotional regulation strategies between athletes who met the EDI-3 RF remission criteria and those who did not, as well as ANOVA tests to compare variables according to the stage of adolescence. Finally, linear regression analyses were performed to examine the associations between emotional suppression, cognitive reappraisal, and ED risk indicators. Standardized and unstandardized coefficients, 95% confidence intervals, and *R*^2^ values were reported to facilitate interpretation.

## 3. Results

The results of the descriptive statistics, reliability, and normality of the scales are shown in Table 1. Skewness and kurtosis were within acceptable ranges for most variables, except for the Bulimia variable (Lloret-Segura et al., 2014). Given these indicators, parametric analyses were considered appropriate. Internal consistency of the scales was acceptable to very good (*ω* = 0.74–0.92).

Table 2 presents the goodness-of-fit indices of the Confirmatory Factor Analysis (CFA) for the ERQ-CA and EDI-3RF scales. CFA was conducted using Maximum Likelihood (ML). The ERQ-CA two-factor model (Cognitive Reappraisal and Expressive Suppression) showed excellent fit, supporting the proposed structure. The correlation between the two factors was moderate and significant (*r* = 0.373; *p* < 0.001), indicating a positive association between these two emotion regulation strategies.

For the EDI-3RF, the three-factor model showed an acceptable fit to the data. Although some fit indices were slightly below conventional thresholds, the overall results support the structural validity of the scale’s three-factor structure. Given the non-normality observed in the Bulimia subscale, an additional CFA using robust maximum likelihood (MLR) was conducted. The results yielded highly similar fit indices (*χ*^2^*/df* = 3.05, CFI = 0.845, NNFI = 0.829, RMSEA = 0.082), confirming the robustness of the structural validity findings. The correlations between factors were strong and significant: Factor 1 with Factor 2 (*r* = 0.83, *p* < 0.001), Factor 1 with Factor 3 (*r* = 0.66, *p* < 0.001), and Factor 2 with Factor 3 (*r* = 0.69, *p* < 0.001), indicating substantial positive associations among the three dimensions. The standardized factor loadings and the CFA path diagram are presented in the online Supplementary Materials.

To examine measurement invariance of the ERQ-CA, multigroup CFAs were conducted across sex and adolescence stages, following the standard sequence of invariance testing: configural → metric → scalar → strict. Table 3 shows the fit indices for these analyses. For sex, the configural invariance model indicated a good fit, supporting a similar factor structure for males and females. Imposing equality constraints on factor loadings (metric invariance), intercepts (scalar invariance), and residuals (strict invariance) resulted in no substantial decrement in fit indices (ΔCFI ≤ 0.01, ΔRMSEA ≤ 0.015), confirming full measurement invariance between sexes.

For adolescence stage, the configural invariance model also showed an acceptable fit, indicating that the basic factor structure was similar across early, middle, and late adolescence. However, imposing equality constraints on factor loadings (metric invariance) led to a noticeable decrease in model fit (ΔCFI = −0.029), suggesting partial metric invariance. Subsequent constraints on intercepts (scalar invariance) resulted in a small improvement in fit (ΔCFI = +0.005), whereas equality of residuals (strict invariance) caused a substantial decrement (ΔCFI = −0.030), indicating that strict invariance was not supported. These results suggest that the ERQ-CA demonstrates partial measurement invariance across adolescence stages, meaning that factor loadings and residuals may vary somewhat by age group, but overall, the scale retains a comparable factor structure.

Regarding correlations between variables (Table 4), emotional suppression correlated positively with ED variables: drive for thinness (*p* < 0.01), body dissatisfaction (*p* < 0.01) and bulimia (*p* < 0.05), suggesting that higher levels of emotional suppression are associated with higher risk indicators in eating behavior. On the other hand, cognitive reappraisal showed negative correlations with clinical variables, although only the association with body dissatisfaction was significant (*p* < 0.05).

Comparisons were performed of emotional regulation strategies among athletes who met the EDI-3 RF referral criterion C and those who did not (Table 5). This criterion is based on the presence of specific behavioral symptoms suggestive of a possible eating disorder, including five behavioral indicators: binge eating episodes, self-induced vomiting, laxative abuse, excessive exercise to control weight, and extreme weight loss. According to the EDI-3 RF manual, individuals meeting the cutoff for at least one of these behavioral symptoms are recommended for referral to specialized treatment. The results showed no significant differences in cognitive reappraisal between the groups. However, in emotional suppression, athletes in the referral group had significantly higher scores compared to those in the non-referral group, with a small effect size (*p <* 0.01; *d* = 0.32).

Regarding comparisons based on age, participants were classified according to the developmental framework proposed by Sawyer et al. (2018) and the American Academy of Pediatrics et al. (2019), which defines adolescence as spanning from 10 to 25 years and distinguishes three stages: early adolescence (10–13 years), middle adolescence (14–17 years), and late adolescence (18–25 years) (Table 6). Significant differences were found in the risk factor for ED body dissatisfaction (*p* = 0.039). To determine between which groups these differences occurred, Scheffé post hoc tests were conducted. The difference between the middle and early adolescence groups approached significance (*t* = 2.40, *p* = 0.057). No significant differences were observed for other ED risk factors or emotion regulation variables.

Finally, linear regression analyses (Table 7) were conducted to examine the associations of cognitive reappraisal and emotional suppression with drive for thinness, body dissatisfaction, and bulimia, while controlling for the C referral criterion of ED (behavioral symptoms). This control ensures that the associations between emotion regulation strategies and eating disorder risk factors are not confounded by differences in referral status. No additional covariates (e.g., age, sex, sport type, training load) were included in these models. The models were significant for all ED risk indicators (*p* < 0.001) and explained between 5% and 14% of the variance in the respective outcomes, with collinearity diagnostics (Tolerance > 0.90, VIF < 1.2, Condition Index < 13) indicating no evidence of problematic multicollinearity among predictors. Emotional suppression was positively associated with higher scores across all outcomes, while cognitive reappraisal showed a consistent negative association with these risk factors. A compact visual summary of these standardized *β* coefficients is presented in the online Supplementary Materials (forest plot).

## 4. Discussion

This study aimed to examine the reliability and structural validity of the ERQ-CA in young Mexican athletes, as well as to analyze the relationship between emotional regulation strategies (ES and CR) and risk factors associated with ED (drive for thinness, body dissatisfaction and bulimia) in this sample. Overall, our hypotheses were partially supported. The ERQ-CA demonstrated the expected two-factor structure and showed measurement invariance across sex, confirming its reliability and applicability in this population. Invariance across adolescence stages was partial, indicating some variation by developmental stage. Invariance across adolescence stages was partial, and configural invariance was supported, indicating that the basic factor structure is comparable across early, middle, and late adolescence; partial metric and scalar invariances were observed, whereas strict invariance was not supported. These results suggest some variation by developmental stage, consistent with previous research on the influence of age and maturation on emotion regulation (Gross, 1998; Tamminen et al., 2025).

The ERQ-CA showed acceptable to very good internal consistency (*ω* > 0.70), consistent with prior validations in international contexts (Aune et al., 2025; Chen et al., 2023; Gong et al., 2022), confirming the instrument’s suitability for young athletes in Latin America. The ease of administration of this brief 10-item tool supports its potential use in routine assessments within the Mexican sports context.

Although the Bulimia subscale showed slight deviations from normality, parametric analyses were applied to maintain consistency across scales. The three-factor structure of the EDI-3RF showed slightly suboptimal fit indices (CFI = 0.839; NNFI = 0.823; RMSEA = 0.085), primarily due to non-normality in the Bulimia subscale, which may limit the precision of factor-level inferences. Robustness checks using robust maximum likelihood (MLR) confirmed highly similar fit indices, supporting the stability of the factor structure and parameter estimates. For all subsequent analyses (correlations, group comparisons, and regression analyses), observed composite scores (scale means) were used to ensure interpretability and consistency across scales. Therefore, associations involving EDI-3RF factors should be interpreted with caution.

Regarding the correlation between emotional regulation strategies and ED risk, emotional suppression was positively correlated with all three risk indicators (drive for thinness, body dissatisfaction and bulimia). This finding is consistent with previous research, identifying ES as a maladaptive tactic associated with greater vulnerability to risky eating behaviors (Brytek-Matera et al., 2022; Mendia et al., 2024; Prefit et al., 2019). On the other hand, cognitive reappraisal showed a negative correlation with body dissatisfaction, supporting its role as a protective factor, although associations with other risk indicators were non-significant. This suggests that a greater ability to reinterpret and re-signify situations in a positive way may work as a preventive factor against disorders such as ED. These results partially confirm our hypothesis that CR would be negatively associated with ED risk and aligns with studies that highlight the benefits of psychological flexibility in promoting healthier eating behaviors and body acceptance (Bluett et al., 2016; Cramer et al., 2018; Pavisich et al., 2024).

Comparisons of emotional regulation strategies in athletes meeting the EDI-3 RF referral criteria versus non-referral athletes showed higher levels of emotional suppression in the elevated-risk group, reinforcing the importance of ES as a potential early marker for DE in sport contexts. These findings are consistent with López and Chicote (2017) and Mercado (2023), who also found that ES is a significant risk factor in adolescent athletes, highlighting the potential usefulness of assessing emotion regulation strategies as a complement for early detection of eating problems in sport settings. CR did not differ between groups, suggesting that while it may protect against body dissatisfaction, its effects may be more subtle or context dependent. This aligns with prior evidence on adaptive emotion regulation in sport (Bird et al., 2021; Boas Junior et al., 2025).

Given partial metric and scalar invariance across adolescence stages, comparisons of latent means should be interpreted with caution, as factor loadings and residuals may vary across stages. Adolescence stages comparisons revealed increases in drive for thinness and body dissatisfaction during adolescence, confirming that adolescence represents a critical period for ED emergence (Crone, 2016; Granz et al., 2019; Parise et al., 2019; Pérez-Sánchez et al., 2024; Slobodskaya, 2021). Post hoc tests suggested the middle-adolescence group may experience the highest vulnerability, consistent with neurodevelopmental and hormonal factors that influence emotion regulation and self-concept at this stage (World Health Organization, 2025; Pan American Health Organisation, 2025).

Linear regressions examined the associations between emotional regulation strategies and ED risk indicators. ES was positively associated with all three ED risk indicators (drive for thinness, body dissatisfaction, and bulimia), suggesting that greater avoidance of emotional expression corresponds with higher preoccupation with weight and shape, as well as a higher likelihood of engaging in harmful eating behaviors. In contrast, CR was negatively associated with body dissatisfaction and bulimia, with the strongest association observed for body dissatisfaction, indicating that a greater ability to reinterpret situations is linked to lower body dissatisfaction and lower likelihood of bulimic behaviors (Arellano Holguín et al., 2021; Rojas Palomino, 2022).

Overall, these results confirm the maladaptive versus protective roles of these strategies, consistent with theoretical models of emotion regulation (Gross, 1998) and empirical studies in athletic populations linking suppression to poorer psychological outcomes and reappraisal to enhanced resilience and performance (Martínez-Líbano et al., 2025; Tamminen et al., 2025). Although the variance explained by these models was modest (5–14%), it remains meaningful given the multifactorial nature of ED and highlights the importance of emotion regulation as one of multiple contributing factors.

### 4.1. Limitations, Future Lines and Practical Applications

This study has several limitations. Its ccross-sectional design prevents the establishment of causal relationships between the variables analyzed. The regression models explained a modest proportion of variance in ED risk indicators, suggesting that other factors beyond emotional regulation, such as psychological, biological, or contextual factors, likely contribute to risk. Additionally, reliance on self-report measures, the lack of clinical interviews or diagnoses, the sample size, and its potential lack of representativeness across different sports disciplines limit the generalizability of the findings.

Although robustness was verified using robust maximum likelihood (MLR) for the Bulimia subscale, additional sensitivity analyses, such as bootstrap confidence intervals or controlling for potential confounding variables (e.g., training hours, participation in weight-sensitive sports), were not conducted. Partial invariance across adolescence stages, together with a slightly suboptimal fit of the EDI-3RF, indicates that comparisons involving latent factors and some factor-level interpretations should be made with caution. These omissions may limit the robustness of the findings and should be addressed in future research.

Moreover, cultural factors specific to Mexican youth sport (such as training practices, competitive pressures, and social norms regarding body image) may constrain the applicability of these results to athletes in other countries or disciplines. For future research, longitudinal studies are suggested to examine the directionality of the relationships and follow-up of the athletes over time. In addition, expanding the sample to include multiple sport disciplines would allow identification of possible higher-risk sports.

The present findings have practical implications for coaches, parents, and mental health professionals. Monitoring athletes’ use of ES and promoting CR may serve as useful adjuncts in psychoeducation and early support within sports settings, enhancing emotional flexibility and potentially reducing ED risk. These recommendations should be considered supportive guidance rather than screening or diagnostic advice, given the modest variance explained and the measurement constraints of the EDI-3RF. In this context, the development and evaluation of interventions aimed at strengthening adaptive emotion regulation strategies, such as CR, is recommended to examine their impact on reducing ED risk indicators in athletes. This approach aligns with prior calls for interventions targeting emotion regulation in high-pressure sport contexts (Tamminen et al., 2025; Berengüí et al., 2024).

### 4.2. Conclusions

This study supports the reliability and structural validity of the ERQ-CA in Mexican young athletes and shows that expressive suppression is positively associated with ED risk indicators, whereas cognitive reappraisal is negatively associated. These findings are consistent with existing emotion regulation theories and indicate that adolescence is an important developmental stage for understanding these associations in athletic populations. They also suggest that monitoring emotion regulation may help inform supportive strategies within adolescent sports contexts.

## Figures and Tables

**Table 1:** Descriptive statistics, skewness, kurtosis, reliability and normality.

	Mean	SD	Skewness	Kurtosis	*ω*
ERQ-CA					
Cognitive reappraisal	3.54	0.76	−0.22	0.14	0.75
Expressive suppression	2.91	0.93	0.02	−0.56	0.74
EDI-3 RF					0.92
Drive for thinness	1.16	1.19	0.83	−0.53	0.88
Body dissatisfaction	1.04	0.82	0.77	0.15	0.82
Bulimia	0.49	0.70	2.29	5.60	0.85

Note. *ω* = McDonald’s omega; ERQ-CA = Emotion Regulation Questionnaire for Children and Adolescents; EDI-3 RF = Eating Disorder Inventory-3 Referral Form.

**Table 2:** Confirmatory Factor Analysis (CFA).

	*χ* ^2^	*df*	*χ* ^2^ */df*	NNFI	CFI	RMSEA
ERQ-CA	37.38	34	1.09	0.993	0.995	0.018
EDI-3 RF	847.75	272	3.11	0.823	0.839	0.085

Note. *χ*^2^ = Chi-squared; *df* = degrees of freedom; NNFI = Bentler–Bonett Non-Normed Fit Index; CFI = Comparative Fit Index; RMSEA = Root Mean Square Error of Approximation.

**Table 3:** Results of multigroup factorial invariance analyses by sex and adolescence stage.

Group/Model	*χ*^2^ (*df*)	*χ* ^2^ */df*	RMSEA (90% CI)	SRMR	∆*χ*^2^ (∆*df*)	CFI (∆CFI)	ECVI	Comparison	Decision
**Sex**									
Configural	78.93 (68)	1.16	0.033 (0.00–0.061)	0.052	–	0.984 (–)	0.552	–	–
Metric	95 (76)	1.25	0.041 (0.00–0.065)	0.070	16.07 (8)	0.972 (−0.012)	0.553	Model 1 vs. Model 2	Accept
Scalar	101.76 (84)	1.21	0.038 (0.00–0.062)	0.066	6.75 (8)	0.974 (+0.002)	0.657	Model 2 vs. Model 3	Accept
Strict	108.16 (94)	1.15	0.032 (0.00–0.056)	0.067	6.40 (10)	0.979 (+0.005)	0.611	Model 3 vs. Model 4	Accept
**Adolescence stage**									
Configural	124.98 (102)	1.22	0.048 (0.00–0.074)	0.066	–	0.968 (–)	0.851	–	Accept
Metric	161.44 (118)	1.36	0.061 (0.035–0.083)	0.093	36.46 (16)	0.939 (−0.029)	0.866	Model 1 vs. Model 2	Reject
Scalar	174.24 (134)	1.3	0.055 (0.028–0.077)	0.088	12.80 (16)	0.944 (+0.005)	1.004	Model 2 vs. Model 3	Accept
Strict	215.47 (154)	1.39	0.064 (0.042–0.083)	0.088	41.23 (20)	0.914 (−0.030)	1.008	Model 3 vs. Model 4	Reject

Note. *χ*^2^ (df) = chi-square and degrees of freedom; *χ*^2^/*df* = relative chi-square; RMSEA (90% CI) = root mean square error of approximation with 90% confidence interval; SRMR = standardized root mean square residual; ∆*χ*^2^ (∆df) = chi-square difference between nested models; CFI (∆CFI) = comparative fit index with change; ECVI = expected cross-validation index.

**Table 4:** Correlation between EDI-3RF and ERQ-CA.

	1	2	3	4	5
1. Cognitive reappraisal	—				
2. Emotional suppression	0.272 ***	—			
3. Drive for thinness	−0.078	0.182 **	—		
4. Body dissatisfaction	−0.126 *	0.164 **	0.722 ***	—	
5. Bulimia	−0.095	0.135 *	0.607 ***	0.616 ***	—

Note. * *p* < 0.05; ** *p* < 0.01; *** *p* < 0.001.

**Table 5:** Differences between athletes in remission and non-remission.

	Referral Group *n* = 160		Non-Referral Group *n* = 135		*t*	*df*	*p*	*d*	95% CI
	*M*	*SD*	*M*	*SD*					
Cognitive reappraisal	3.54	0.78	3.54	0.74	0.00	293	0.996	0.00	[−0.22, 0.23]
Emotional suppression	3.05	0.92	2.75	0.91	−2.77	293	**0.006**	0.32	[−0.55, −0.09]

Note. *t* = t statistic; *df* = degrees of freedom; *p* = significance level; *d* = Cohen’s d (effect size); 95% CI = 95% confidence interval for the mean difference. *S*tatistically significant *p*-values are bolded. In the EDI-3 RF manual, this criterion is referred to as “remission.” However, in this study it refers to a screening-based threshold indicating the need for referral to specialized clinical evaluation and does not imply a clinical diagnosis or psychiatric remission.

**Table 6:** Differences in Emotion Regulation and ED scales by adolescence stage.

	Adolescence						*F*	*p*	*η* ^2^	95% CI
	Early		Middle		Late					
	*n* = 62		*n* = 93		*n* = 140					
	*M*	*SD*	*M*	*SD*	*M*	*SD*				
Cognitive reappraisal	3.59	0.76	3.57	0.85	3.50	0.71	0.40	0.668	0.003	[0.00, 0.02]
Emotional suppression	2.79	0.93	2.98	0.96	2.91	0.90	0.81	0.443	0.006	[0.00, 0.02]
Drive for thinness	0.96	1.20	1.35	1.23	1.12	1.16	2.04	0.131	0.014	[0.00, 0.04]
Body dissatisfaction	0.81	0.89	1.13	0.76	1.08	0.80	3.27	**0.039**	0.022	[0.00, 0.06]
Bulimia	0.49	0.81	0.52	0.65	0.48	0.68	0.11	0.891	0.000	[0.00, 0.01]

Note. *F* = F statistic from ANOVA; *p* = significance level; *η*^2^ = eta-squared (effect size); 95% CI = 95% confidence interval for the effect size. *S*tatistically significant *p*-values are bolded.

**Table 7:** Associations between emotional regulation and ED risk indicators.

Model	*R*	*R* ^2^	Adjusted *R*^2^	RMSE	*F*	*p*	Predictor	*B*	*SE*	*β*	95% CI	*p*
Drive for thinness	0.224	0.050	0.044	1.170	7.746	<0.001	CR	−0.20	0.09	**−0.12**	[−0.37, −0.02]	0.029
							ES	0.23	0.07	**0.18**	[0.09, 0.38]	0.002
Body dissatisfaction	0.350	0.122	0.113	0.772	13.54	<0.001	CR	−0.18	0.06	**−0.17**	[−0.30, −0.06]	0.003
							ES	0.15	0.05	**0.17**	[0.05, 0.25]	0.004
Bulimia	0.377	0.142	0.133	0.653	16.03	<0.001	CR	−0.11	0.05	**−0.12**	[−0.21, −0.01]	0.026
							ES	0.08	0.04	**0.11**	[0.00, 0.17]	0.041

Note. *R* = Multiple correlation; *R*^2^ = Coefficient of determination; RMSE = Root mean square error; CR = Cognitive Reappraisal; ES = Emotional Suppression; *B* = unstandardized coefficient; SE = standard error; *β* = standardized coefficient. Standardized coefficients with statistically significant *p*-values are bolded.

## Data Availability

The analysis scripts for the confirmatory factor analyses (CFAs) and regression models are available from the corresponding author upon reasonable request. Raw data cannot be shared due to participant confidentiality and ethical restrictions. Data may be obtained from the corresponding author.

## Supplementary Materials

The following supporting information can be downloaded at https://www.mdpi.com/article/10.3390/ejihpe15090188/s1, Figure S1: CFA Path Diagram EDI-3RF; Figure S2: CFA Path Diagram ERQ-CA; Figure S3: Forest Plot of standardized *β* coefficients.
