# Supplementary material for: Emotional Regulation and Risk of Eating Disorders in Adolescent Athletes
Source: Eur J Investig Health Psychol Educ. 2025 Sep 18;15(9):188. doi: 10.3390/ejihpe15090188 (PMC12468510; doi:10.3390/ejihpe15090188)
Supplement: Supplementary file 1 [file ejihpe-15-00188-s001.zip › ejihpe-3779894-supplementary.pdf]

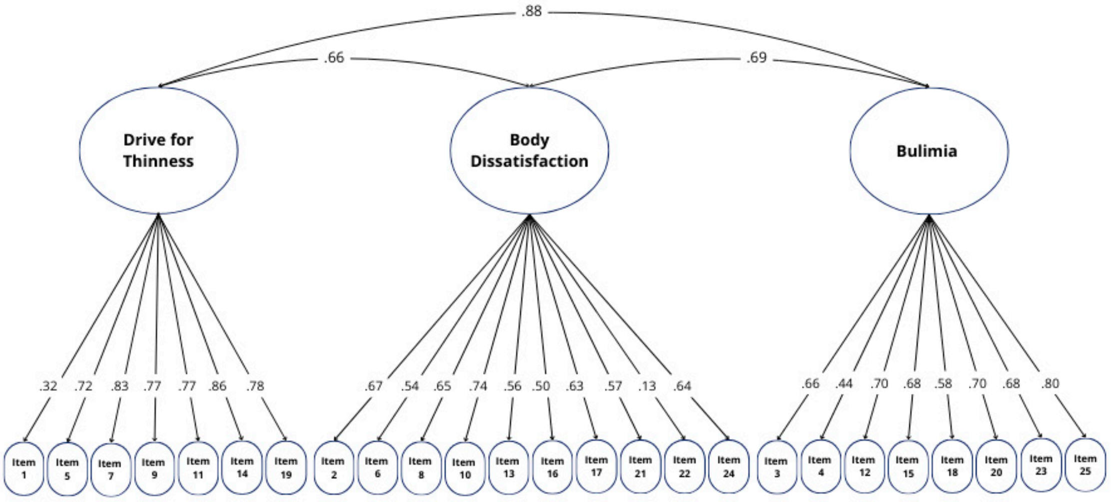

Figure S1. CFA Path Diagram EDI-3RF.

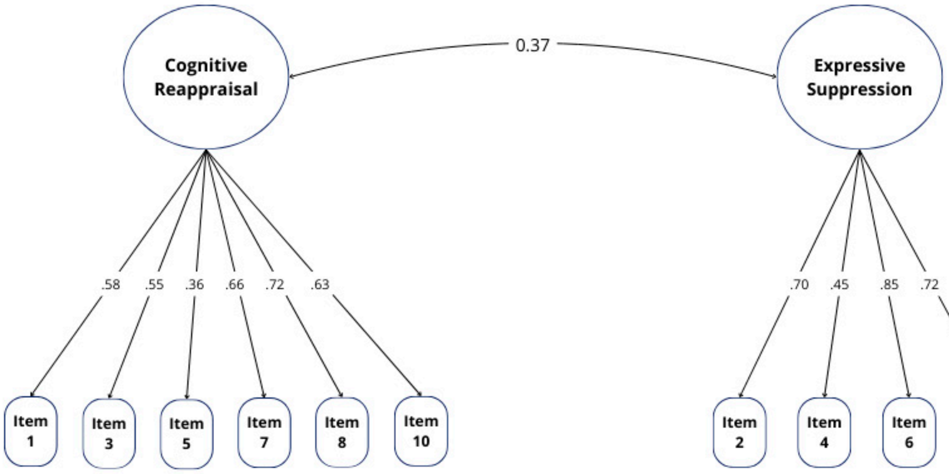

Figure S2. CFA Path Diagram ERQ-CA.

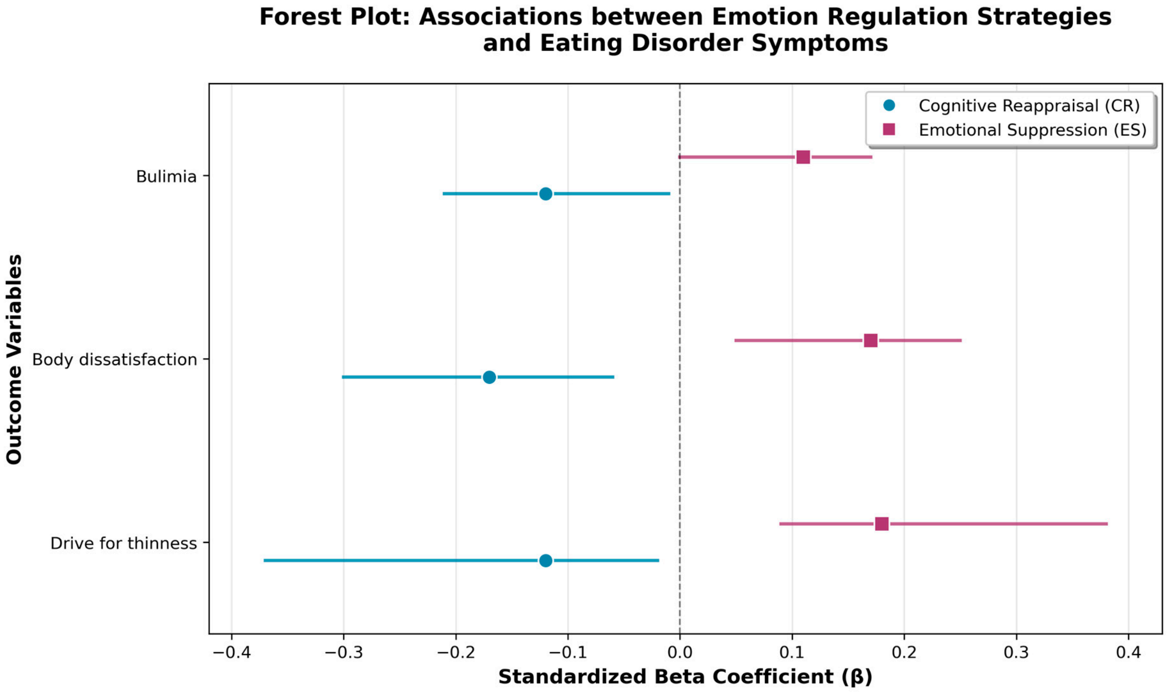

Figure S3. Forest Plot of standardized β coefficients.
